# A systematic review of LGBTQ+ identities and topics in sport leadership

**DOI:** 10.3389/fspor.2024.1414404

**Published:** 2024-07-01

**Authors:** Colleen S. O’Connell, Anna Bottino

**Affiliations:** ^1^ Department of Wellness and Sport Sciences, Millersville University, Millersville, PA, United States; ^2^ Department of Exercise Science and Athletic Training, Springfield College, Springfield, MA, United States

**Keywords:** LGBTQ, coach, leader, administrator, sport, sexuality, leadership

## Abstract

**Introduction:**

As individuals with occupational status and power, sport leaders (e.g., coaches and athletic administrators) are responsible for enforcing cultures of inclusion within institutions of athletics. Yet, sport leaders who possess LGBTQ+ sexual identities are frequently marginalized and stigmatized by entities within and outside of athletics (e.g., athletes, parents of athletes, colleagues). Therefore, LGBTQ+ sport leaders are often faced with a challenging set of circumstances: negotiate the authenticity of their sexual orientation in the context of sport, or leave the profession entirely.

**Methods:**

The purpose of this study was to conduct a systematic review of research related to LGBTQ+ sport leader experiences. Using the Preferred Reporting Items for Systematic Reviews and Meta-Analyses (PRISMA), research across six countries (China/Taiwan/Hong Kong, Italy, New Zealand, Norway, United Kingdom, United States) between 1997 and 2021 was analyzed.

**Results:**

Themes across included studies (*N *= 34) describe intrapersonal experiences of LGBTQ+ sport leaders, interpersonal studies examining stakeholder attitudes (i.e., parents and athletes) toward LGBTQ+ sport leaders, and sport manager attitudes toward LGBTQ+ topics.

**Discussion:**

Findings convey that sport leaders continue to face marginalization due to the presence of heterosexism and heteronormativity in athletics. Future research should continue to explore LGBTQ+ sport leader experiences, behaviors, attitudes, and identities to determine their impact on fostering inclusion and belonging within athletic spaces.

## 1 Introduction

Research on lesbian, gay, bisexual, transgender, and queer (LGBTQ+^1^) identities in sport has expanded tremendously over the previous decade (1). Reviews of LGTBQ+ scholarship in sport-related disciplines have identified a wide range of topic areas that have been examined, including athlete experiences and identities; policy, management, and advocacy; and experiences of sexual prejudice, discrimination, and homophobia among others (1–3). Notably, an understudied area within this scholarship regards the experiences of LGBTQ+ sport leaders, including coaches and athletic administrators (1, 4).

Although there are some indications of LGBTQ+ experiences in and across sport improving [e.g., increased prevalence of athletes coming out and promoting LGBTQ+ social justice initiatives (5–8)], LGBTQ+ sport leaders continue to report marginalization and stigmatization in the context of sport (9, 10). For instance, LGBTQ+ coaches and administrators encounter discrimination on an everyday basis from athletes, parents, colleagues, and other stakeholders (11–13). These discriminatory behaviors can be overt in nature, consisting of homophobic comments (14) or negative recruitment strategies [e.g., “gay bashing” (15)]. Discrimination can also occur through covert actions, such as the lack of intervention when LGBTQ+ individuals encounter homophobic remarks (16), or the avoidance of discussing LGBTQ+ identities [e.g., “Don't Ask, Don't Tell” attitudes (13, 17, 18)]. Whether overt or covert in nature, this discrimination is rooted in heterosexism, a system of attitudes and beliefs carried out through structural practices and interpersonal behaviors to reinforce heterosexuality as the norm [i.e., heteronormativity (19)]; thereby labeling LGBTQ+ individuals as “other” and “deviant” (20).

Ultimately, encountering heterosexism at a structural level and stigmatization and/or discrimination at an interpersonal level has led to many LGBTQ+ sport leaders leaving the profession (15, 21), or negotiating their identities in the workplace (20, 22). These negotiations include: dressing or acting in a stereotypically feminine (16) or masculine (23) manner, concealing or compartmentalizing personal lives from professional lives (13, 24), and prioritizing professional identities over sexual orientation (25). These strategies enable sport leaders to successfully navigate their occupational environments in light of their marginalized sexual orientation.

Sport leaders uphold a variety of occupational responsibilities; broadly, they oversee the implementation of policies and practices within their organizations, athletic departments, and/or teams (26). They also possess the status and power to influence organizational culture related to diversity, equity, and inclusion [DEI; (27)]—especially by what they say or fail to say in relation to DEI topics, issues, and initiatives (18, 28, 29). Because sport leaders retain occupational status and power in their respective roles and organizations, it is necessary to explore potential resistances and opportunities for action related to LGBTQ+ topics (7, 30). Thus, developing a holistic understanding of existing research is critical, especially as it pertains to the experiences of LGBTQ+ sport leaders, and sport leaders’ attitudes regarding LGBTQ+ issues.

The purpose of this study was to conduct a systematic review of research related to LGBTQ+ sport leader experiences, stakeholder attitudes toward current or former LGBTQ+ sport leaders, and the attitudes of sport leaders toward LGBTQ+ issues using the Preferred Reporting Items for Systematic Reviews and Meta-Analyses (PRISMA). The current study responds to calls for additional research on LGBTQ+ topics for sport leaders, including coaches, administrators, and managers (1, 3, 4). By critically examining previous scholarship, this systematic review provides next steps for research related to LGBTQ+ sport leaders and LGBTQ+ inclusive leadership practices.

## 2 Methods

### 2.1 Search process

A systematic review was conducted by searching six databases (SPORTDiscus, PsycINFO, Business Source Complete, ERIC, SocINDEX, and Academic Search Complete). Databases were selected based on their alignment with the topic area for this study (i.e., SPORTDiscus, PsycINFO, Business Source Complete, ERIC, SocINDEX) and their breadth of scholarly research (i.e., Academic Search Complete) in order to ensure relevant scholarship was included. The following keyword combinations were used: ““gay or lesbian or bisexual or homosexual or “same sex” or transgender or queer or GLBT or LGBT or LGBTQ or LGBTQ+”” AND ““sport or athletics or team or basketball or soccer or lacrosse or swimming or diving or track or “track and field” or volleyball or “field hockey” or hockey or wrestling or gymnastics or golf or tennis or football or crew or fencing or softball or baseball or rugby”” AND “management or manager or director or administrator or administration or coach* or sport coach*”. Articles were also hand-searched to include relevant studies not found in the initial search process, resulting in the addition of three references. The original search process took place between September and November of 2021. The search was subsequently updated in September of 2022 and May of 2024 to confirm no new scholarship meeting inclusion criteria had been published since the original search. Both updated searches did not yield any scholarship that met inclusion criteria for this study.

**Figure 1:**
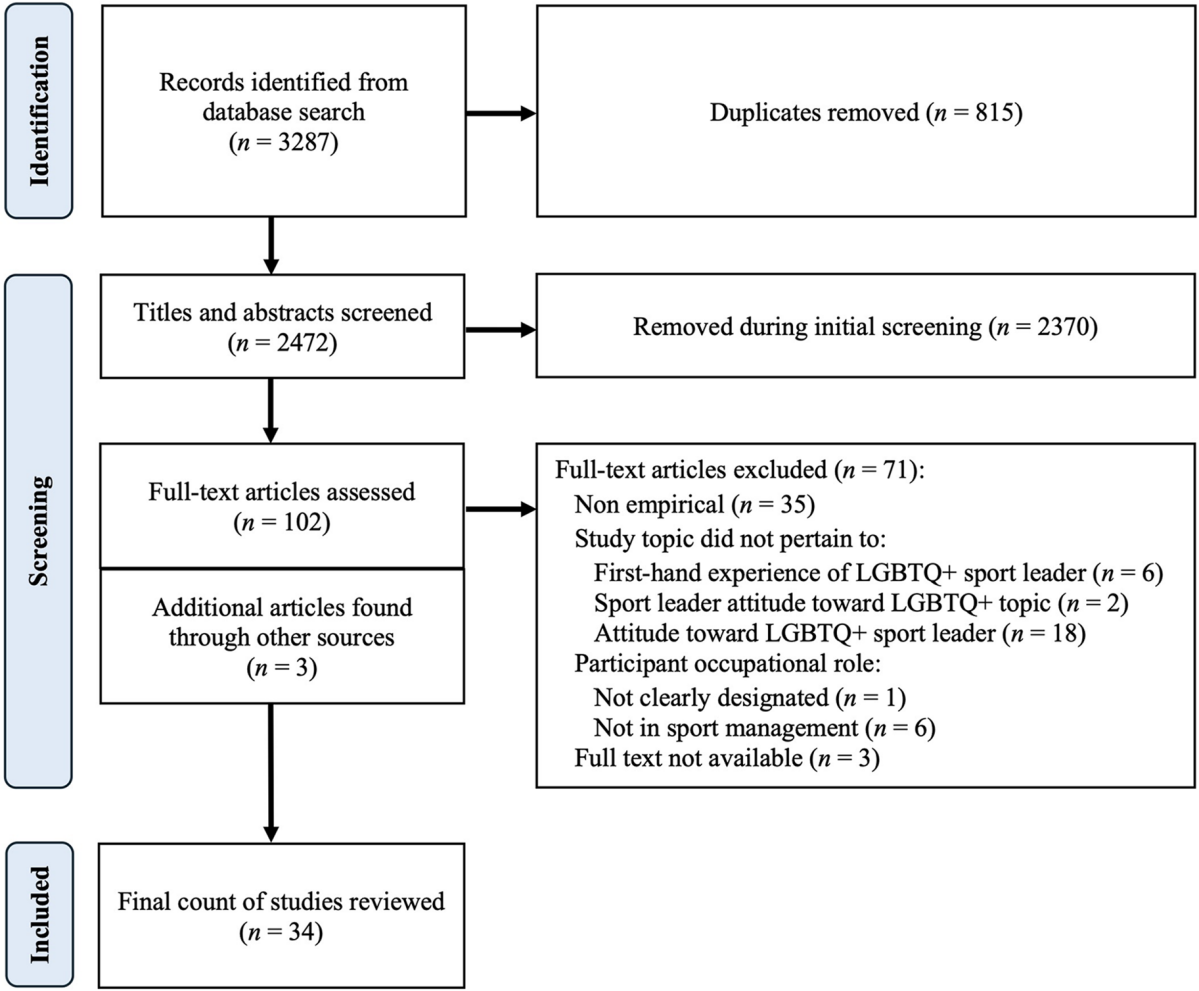
PRISMA flow diagram of study selection process.

Articles were included based on the following criteria: (a) the study was an original empirical study; (b) the study topic pertained to (i) first-hand, lived experiences reported by LGBTQ+ individuals working in managerial roles in sport; or (ii) sport stakeholder attitudes toward LGBTQ+ individuals that occupy managerial roles in sport; or (iii) attitudes of individuals working in sport managerial roles toward LGBTQ+ issues within sport; (c) the managerial role in the sport organization was designated as athletic director, athletic administrator, sport manager, sport information director, support staff, or sport coach. To reduce the possibility of missing relevant studies, there was no date restriction for the search process; any record published within searched databases up to and including the date of original and updated search(es) was screened. References that were excluded during the screening process included: (a) media or journalistic reports, textbook chapters, and non-empirical studies; (b) studies in which (i) all participants were heterosexual or sexual orientation was not designated; or (ii) topics other than attitudes towards occupational role designee were explored; or (iii) LGBTQ+ physical or mental health behaviors or issues were researched; (c) studies in which participant roles were not clearly designated or roles were not in the sport industry. The selection process was conducted in four phases according to PRISMA guidelines and is displayed in Figure 1 (31).

### 2.2 Quality appraisal

Included articles did not contain studies with randomized control trials; overall, the studies utilized a variety of methodologies to conduct empirical research. A quality assessment of bias was performed using two accepted standards of methodological appraisal. For quantitative studies, risk of bias assessments were informed by the U.S. Preventive Services Task Force (USPSTF) observational research criteria tool as shown in Table 1 (43); for qualitative studies, the Critical Appraisal Skills Programme (CASP) was utilized, as shown in Table 2 (55, 56). Mixed methods studies were evaluated through both standards of appraisal. Both authors independently completed quality assessments for all included articles. Then, authors jointly discussed any discrepancies regarding methodological rigor of included studies until reaching consensus regarding if and how each study met appraisal criteria. Following quality assessment, relevant information was extracted from selected articles. Extracted information, displayed in Table 3, included country of study, methodological design, theoretical framework, subject focus, sample (participant characteristics), and results.

**Table 1:** Quality assessment of quantitative research.

Study	Item 1	Item 2	Item 3	Item 4	Item 5	Item 6	Number of criteria met	Number of criteria to meet
Amodeo et al. (32)	1	0	1	0	1	1	4	6
*Bass et al. (33)	1	0	1	N/A	N/A	1	3	4
*Calhoun et al. (34)	1	0	1	N/A	N/A	N/A	2	3
Cunningham and Melton (11)	1	1	1	1	1	1	6	6
Halbrook and Watson (35)	1	0	1	0	1	1	4	6
Hardin et al. (36)	1	1	1	0	0	1	4	6
*Kamphoff (15)	1	0	1	0	1	N/A	3	5
*LaVoi and Glassford (37)	1	0	1	N/A	N/A	N/A	2	3
Mullin and Cook (38)	1	1	1	1	1	1	6	6
Oswalt and Vargas (39)	1	0	1	0	1	1	4	6
Sartore and Cunningham (40)	1	0	1	0	0	1	3	6
*Sartore and Cunningham (10)	1	0	1	N/A	0	1	3	5
*Scheadler et al. (41)	1	0	1	0	0	1	3	6
Vargas-Tonsing and Oswalt (42)	1	0	1	0	1	1	4	6

* Mixed Methods Study; Item 1 = Study participants defined (time, place, personal characteristics) [1]; Item 2 = Selection random [1] or consecutive [0]; Item 3 = Standardized validated questionnaire OR clear description of outcomes measured [1]; Item 4 = Participant rate >80% OR if the participant rate is low, comparison respondents/nonrespondents described [1]; Item 5 = Disclosure of ethical review [1]; Item 6 = Inclusion of significant and non-significant data AND appropriate interpretation of statistical results [1].

**Table 2:** CASP checklist for qualitative research appraisal.

Study	Item 1	Item 2	Item 3	Item 4	Item 5	Item 6	Item 7	Item 8	Item 9	Item 10
*Bass et al. (33)	Y	Y	Y	Y	Y	Y	Y	N	Y	Y
Brookens (44)	Y	Y	Y	Y	Y	N	N	N	Y	Y
Calhoun (45)	Y	Y	Y	Y	Y	Y	Y	Y	Y	Y
Cavalier (24)	Y	Y	Y	Y	Y	N	N	Y	Y	Y
Cunningham (46)	Y	Y	Y	Y	Y	Y	Y	Y	Y	Y
Cunningham and Melton (47)	Y	Y	Y	Y	Y	Y	Y	Y	Y	Y
Halbrook et al. (17)	Y	Y	Y	Y	Y	Y	Y	Y	Y	Y
Iannotta and Kane (22)	Y	Y	Y	Y	Y	Y	N	N	Y	Y
*Kamphoff (15)	Y	Y	Y	Y	Y	Y	Y	Y	Y	Y
Kauer (48)	Y	Y	Y	Y	Y	Y	Y	Y	Y	Y
Krane and Barber (16)	Y	Y	Y	Y	Y	Y	Y	Y	Y	Y
*LaVoi and Glassford (37)	Y	Y	Y	Y	Y	Y	Y	Y	Y	Y
MacCharles and Melton (23)	Y	Y	Y	Y	Y	Y	Y	Y	Y	Y
Melton and Cunningham (25)	Y	Y	Y	Y	Y	Y	Y	Y	Y	Y
Melton and Cunningham (49)	Y	Y	Y	Y	Y	Y	Y	Y	Y	Y
Norman (13)	Y	Y	Y	Y	Y	Y	N	Y	Y	Y
Norman (14)	Y	Y	Y	Y	Y	N	N	Y	Y	Y
*Sartore and Cunningham (10)	Y	Y	Y	Y	Y	N	Y	Y	Y	Y
*Scheadler et al. (41)	Y	Y	Y	Y	Y	N	N	Y	Y	Y
Shaw (50)	Y	Y	Y	Y	Y	Y	Y	Y	Y	Y
Skogvang and Fasting (51)	Y	Y	Y	Y	Y	N	Y	Y	Y	Y
Tseng and Sum (52)	Y	Y	Y	Y	Y	N	Y	Y	Y	Y
Walker and Melton (21)	Y	Y	Y	Y	Y	Y	Y	Y	Y	Y
Wellman and Blinde (53)	Y	Y	Y	Y	Y	N	Y	Y	Y	Y
Wolf-Wendel et al. (54)	Y	Y	Y	Y	Y	N	N	Y	Y	Y

* Mixed Methods Study; Item 1: Was there a clear statement of the aims of research?; Item 2: Is a qualitative methodology appropriate?; Item 3: Was the research design appropriate to the aims of the research?; Item 4: Was the recruitment strategy appropriate to the aims of the research?; Item 5: Was the data collected in a way that addressed the research issue?; Item 6: Has the relationship between the researcher and the participants been adequately considered?; Item 7: Have ethical issues been taken into consideration?; Item 8: Was the data analysis sufficiently rigorous?; Item 9: Is there a clear statement of findings?, Item 10: How valuable is the research?

Y, yes; N, no; U, unclear.

**Table 3:** Profiles of selected research studies.

Author	Country	Subject focus	Sample	Methodological design	Theoretical framework	Results
Amodeo et al. (32)	Italy	Attitudes of sexual prejudice toward sexual minority athletes	Sport directors and coaches (*n* = 178)	Quantitative (Survey)	Sexual prejudice theory	While sexual prejudice attitudes were low on average, negative attitudes toward LGB athletes was primarily enacted through sport directors and coaches’ denial of visibility of sexual identity in sporting spaces.
Bass et al. (33)	USA	Prevalence of public sexuality in coaching biographies & attitudes towards open homosexuality	College coach biographies from DI FBS colleges; 5 college coaches (*n* = 1,052)	Mixed methods (Descriptive research & interviews)	Framing theory; Sexual prejudice theory	1 coach out of 1,052 biographies listed a same-sex partner. Coaches did not discuss sexuality with players and feared retribution if sexuality was public.
Brookens (44)	USA	NCAA athletic administrator attitudes towards transgender student-athletes inclusion	NCAA athletic administrators (*n* = 511)	Mixed methods (Survey & content analysis)	Queer feminist theory	Athletic administrators demonstrated a lack of education related to transgender student-athlete inclusion. Policymaking was done on an *ad hoc* basis.
Calhoun (45)	USA	Attitudes and gatekeeping behaviors toward online LGBTQ+ coach biographies	DI women's basketball sports information directors (SIDs; *n* = 14)	Qualitative (Critical discourse analysis & interviews)	Gatekeeping theory	SIDs employed gatekeeping practices to exclude same-sex family narratives in LGBTQ+ coaching biographies.
Calhoun et al. (34)	USA	Presence of heteronormative frames in intercollegiate coach online biographies	College coach online biographies from DI and DIII colleges (*n* = 1,855)	Mixed methods (Descriptive research & content analysis)	Framing theory	Six non-heteronormative biographies were observed. DI coaches were more likely to include family narratives than DIII counterparts. Male head coaches were more likely to have family narratives published online.
Cavalier (24)	USA	Gay men's occupational experiences working in sport	Gay men working in sport (*n* = 10)	Qualitative (interviews)	Symbolic interaction framework	Gay men in sport demonstrated active and passive strategies for coming out in sport. They also possessed mixed experiences in their perception of the workplace, disclosure of sexual identity, and anxiety surrounding working in or around the locker room.
Cunningham (46)	USA	Employee attitudes & conceptualization of LGBT inclusiveness	DIII athletic departments (*n* = 2)	Qualitative (Collective case study)	Multilevel framework	Various multilevel factors (individual, organizational, macro) worked in synergy to foster a LGBT-inclusive workplace, which had subsequently allowed coaches and athletic administrators to act as their whole selves at work.
Cunningham and Melton (11)	USA	Sexual prejudice towards LGB athletic coaches	Employees from 4 public universities in the Southwest US (*n* = 238)	Quantitative (Survey)	Sexual stigma and prejudice theory	Antecedents to sexual prejudice are socially constructed and can be moderated by racial background.
Cunningham and Melton (47)	USA	Parents’ positive attitudes towards LGBT coaches	Parents from Southwest US (*n* = 10)	Qualitative (Semi-structured interviews)	Dual attitudes model; multilevel framework	Overall, parents expressed positive attitudes towards LGBT coaches; however, the nature of their support differed and thus was classified into three categories: indifferent towards, qualified, or unequivocal.
Halbrook and Watson (35)	USA	High school coach self-perception of efficacy in coaching LGB athletes	High school coaches (*n* = 631)	Quantitative (Survey)	Not clearly mentioned	Coaches aged 18–29 years old perceived themselves to be more effective in coaching LGB athletes than coaches aged 50 years and older. Coaches with no religious affiliation had higher efficacy scores than coaches with a Baptist affiliation. Coaches who have worked with at least 3 LGB athletes in the past perceived themselves to be most effective.
Halbrook et al. (17)	USA	High school coach attitudes and experiences in coaching openly LGB athletes	High school coaches (*n* = 10)	Qualitative (Semi-structured interviews)	Phenomen-ology	Most coaches felt that sport should be a space free of sexuality, where sexual orientation can be recognized, but not openly discussed; rather, an athlete’s athletic identity as a teammate was described to be more important
Hardin et al. (36)	USA	Sport information directors’ attitudes towards gender issues and homophobia in collegiate sport	DI FBS sports information directors (*n* = 272)	Quantitative (Phone survey)	Gatekeeping theory	The majority of SIDs believed that homophobia was not a problem in men’s or women’s sports. Only one-third of participants agreed that LGB coach family narratives should be included within their coaching biographies.
Iannotta and Kane (22)	USA	Lesbian college coaches’ experiences of sexual identity performance	Lesbian DIII coaches (*n* = 12)	Qualitative (Semi-structured interviews)	Not clearly mentioned	Coaches’ sexual identity formation, performance, and management was fluid. Coaches used radical normalization to confront stigmatization.
Kamphoff (15)	USA	Experiences that shaped former coaches’ decisions to leave profession	Female former coaches surveyed (*n* = 121) and interviewed (*n* = 6)	Mixed methods (Survey & interviews)	Theory of feminism; Bargaining with patriarchy framework	Female coaches left the profession due to lack of support by administration, family commitments, and burnout. Lesbian former coaches hid their sexuality.
Kauer (48)	USA	Lesbian coaches’ experiences of being explicitly out in college athletics; how sexual identity disrupts sport norms	Female lesbian college coaches (*n* = 8)	Qualitative (Semi-structured interviews)	Queer feminist theory	Sexual identity is negotiated differently depending on personal, interpersonal, and environmental contexts. Identity narratives enable agency for disruption of heterosexist norms.
Krane and Barber (16)	USA	Lesbian college coaches’ experiences, identity negotiations, and behaviors in context of sport	Female lesbian college coaches (*n* = 13)	Qualitative (Semi-structured interviews)	Social identity perspective	Coaches encountered both overt and concealed homonegative behaviors within athletic departments resulting in identity management. Coaches served as social change agents in subtle and overt ways.
LaVoi and Glassford (37)	USA	LGBTQ+ coaches’ self-perception of identity as explicitly “out” in college sport	Women's sport head coach online biographies from DI colleges (*n* = 3,601)	Sequential mixed methods (Descriptive research & interviews)	Critical feminist perspective; Identity performance theory	18 total same-sex family narratives were found, accounting for 0.5% of the sample. Coaches managed identities and stigmas on a consistent, transparent, and authentic basis to act in radical normalization within the context of sport.
MacCharles and Melton (23)	USA	Gay men's occupational experiences working in sport as shaped by their individual life events	Gay men working in the sport industry (*n* = 12)	Qualitative (semi-structured interviews & life course mapping)	Sexual stigma and prejudice theory; Life course theory	Sexuality remains stigmatized in sport. Gay men working in sport managed their identities so that their professional and personal lives did not intersect. Allies, inside their organization or within professional networks, positively impacted participants’ career retention and trajectory.
Melton and Cunningham (25)	USA	LGBT sport employees’ social and sexual identities and subsequent influence on occupational experiences	DI Athletic department employees (*n* = 9)	Qualitative (Case study)	Social categorization framework	Employees demonstrated a stronger commitment to their occupational identity rather than their sexual identity within the self-categorization process.
Melton and Cunningham (64)	USA	Exploration of factors that influence sport employee support for LGBT inclusion in organizations	DI athletic department employees (*n* = 13)	Qualitative (interviews, document analysis, observations)	Multilevel framework	A variety of factors, ranging from individual to organizational levels, work synergistically to influence the experiences of LGBT sport employees and subsequent LGBT inclusivity within athletic departments.
Mullin and Cook (38)	USA	College coach attitude toward gay men or lesbian women	College coaches (*n* = 290)	Quantitative (Survey)	Not clearly mentioned	Male coaches displayed more negative attitudes (sexual prejudice attitudes) toward lesbian and gay individuals than female coaches did; although overall, both male and female coaches’ attitudes were generally positive.
Norman (13)	UK	Lesbian sport coaches’ everyday experiences within sport in relation to their sexual and gender identities	Lesbian coaches (*n* = 10)	Qualitative (Semi-structured interviews)	Theory of everyday gendered homophobia	Everyday gendered homophobia towards lesbian coaches is reproduced through problematization, marginalization, and a repression of resistance.
Norman (14)	UK	Lesbian sport coaches’ everyday experiences of gendered homophobia in relation to sport organizational structures	Lesbian coaches (*n* = 10)	Qualitative (Semi-structured interviews)	Theory of everyday gendered homophobia	Sport organizational structures upheld marginalization and silencing of LGBTQ+ sexual identities.
Oswalt and Vargas (39)	USA	College coach levels of heterosexism and attitudes towards gay, lesbian, and bisexual individuals	College coaches (*n* = 289)	Quantitative (Survey)	Not clearly mentioned	Coaches had moderately positive attitudes towards GLB individuals with no differences observed for gender, age, or gender coached.
Sartore and Cunningham (40)	USA	Former athletes’ affinity for teams led by gay or lesbian coaches	Former athletes (*n* = 228)	Quantitative (Survey)	Not clearly mentioned	Male former athletes are more likely to be influenced in terms of their affinity toward a team that is coached that by a gay or lesbian coach than female former athletes.
Sartore and Cunningham (10)	USA	Former and current athlete and parent attitudes towards gay and lesbian sport coaches	Former and current athletes (*n* = 228); University staff members (*n* = 76)	Mixed methods (Survey & content analysis)	Compulsatory sexuality framework	Male and female athletes and parents exhibited sexual prejudice towards both gay and lesbian sport coaches.
Scheadler et al. (41)	USA	Attitudes of sexual prejudice toward prospective coaches	NCAA student-athletes (*n* = 143)	Mixed Methods (Survey & thematic analysis)	Sexual prejudice theory	Student-athletes regarded gay, LGBT-ally, and non-identified coaches higher than anti-LGBT coaches. Some participants viewed revelation of sexual identity as a weakness or questioned its importance, underscoring contradictions in perception of leaders related to LGBT topics.
Shaw (50)	New Zealand	Sport organizations’ approach to implementing anti-homophobia practices	National sport organization representative (*n* = 6)	Qualitative (Semi-structured interviews)	Social identity theory; Critical theory; Poststructuralism	Representatives utilize multiple sources of information to develop inclusive policies. The prioritization of information differed for each organization.
Skogvang and Fasting (51)	Norway	Coach attitudes towards sexuality in sport	Sport coaches (*n *= 8)	Qualitative (Interviews)	Theory of hegemonic masculinity; Concept of symbolic power	Coaches acknowledged less homonegativity towards lesbians (compared to gay men) in football. However, they also demonstrated a lack of acknowledgement of acceptance for lesbians within their clubs.
Tseng and Sum (52)	China, Taiwan, Hong Kong	College coach attitudes toward gay and lesbian athletes	Sport coaches (*n* = 14)	Qualitative (Interviews)	Not clearly mentioned	Sociocultural exposure to LGBT advocacy and experience with LGBT athletes positively impacted coach attitudes toward gay and lesbian athletes. Most coaches did not explicitly recognize gender or sexual identities of athletes.
Vargas-Tonsing and Oswalt (42)	USA	College coach perception of efficacy in coaching gay, lesbian, or bisexual athletes	DI college coaches (*n* = 296)	Quantitative (Survey)	Not clearly mentioned	Coaches demonstrated the strongest belief in their ability to create a respectful environment for their student-athletes, without mentioning sexual identity as an aspect of their athlete's identity. Coaches felt least effective in identifying and leveraging materials related to sexual identity inclusion to share with their athletes or integrate in their coaching.
Walker and Melton (21)	USA	Sport employees’ experiences working in sport in context to their multiple marginalized identities	Former and current intercollegiate sport employees (*n* = 15)	Qualitative (interviews)	Intersectionality; Feminist standpoint theory; Black feminist theory	Coaches and administrators with multiple marginalized identities faced greater pressure to negotiate and manage these identities within intercollegiate athletics, leading them to eventually leave their occupations.
Wellman and Blinde (53)	USA	Female college coaches’ experiences related to the lesbian label and homophobia	DI Women's Basketball head coaches (*n* = 10)	Qualitative (interviews)	Not clearly mentioned	The lesbian label attached to female college coaches negatively impacted them in their profession through hiring practices, career choices, coaching behaviors, and recruitment strategies.
Wolf-Wendel et al. (54)	USA	College coach and administrator attitudes towards non-normative sexualities in sport	DI college athletic departments (*n* = 5)	Qualitative (Comparative case studies)	Not clearly mentioned	Coaches possessed negative attitudes toward gays and lesbians or avoided discussing the topic altogether.

## 3 Results

The initial literature search, conducted by the primary author, resulted in 3,287 articles. After removing duplicate articles by hand, the primary author screened 2,472 articles based on title and abstract relevance, deleting 2,370 articles within the second phase. A total of 102 full-text articles were then assessed according to the three inclusion/exclusion criteria. As discussed previously, both authors independently evaluated all articles and ultimately reached consensus regarding articles included in the final analysis. Thus, 71 articles were removed and 3 additional articles were added, yielding 34 articles to be included in the final analysis (Figure 1).

### 3.1 Profile of selected articles

Empirical studies were published within a 24-year range from 1997 to 2021, with most articles (*n* = 26) being published after 2010. Reviewed studies were predominantly composed of samples from the US (*n* = 28), with additional studies containing samples from the United Kingdom (*n* = 2), China/Taiwan/Hong Kong (*n* = 1), New Zealand (*n* = 1), Norway (*n *= 1), and Italy (*n* = 1). Additionally, ten authors accounted for multiple articles (*n* = 18).

### 3.2 Study design, data analysis, and quality appraisal

Selected studies possessed a variety of methodological designs, including mixed methods (*n* = 7), quantitative (*n* = 8), and qualitative (*n* = 19). Mixed method designs most frequently used survey research (*n* = 4) for quantitative analysis, while interviews (*n* = 3) and content analysis (*n* = 3) were most used in qualitative analyses. All quantitative studies used survey research (*n* = 8); qualitative studies used interviews (*n* = 16) and case studies (*n* = 3).

Two quantitative studies met all appraisal criteria (11, 38), while two quantitative studies met all but one aspect of appraisal criteria (Table 1). Remaining quantitative studies (*n* = 10) met most of the appraisal criteria with scores of 3/6, 3/5, or 4/6. Because of the stigmatized subject of articles, random selection of participants (Item #2; Table 1) was not frequently used by researchers and participant rates were generally low. However, these articles (*n* = 10) were deemed to be of high enough quality for inclusion, as all studies defined participants, described significant and non-significant results appropriately, and used a validated questionnaire or clearly described measured outcomes.

Twelve qualitative studies met all appraisal criteria, as displayed in Table 2. Remaining studies (*n* = 13) met most appraisal criteria, including statement of aims, appropriate methodology and research design, defined recruitment strategy, relevant data collection, rigorous data analysis, and statement of findings. However, 36% of qualitative studies (*n* = 9) did not delineate the researcher-participant relationship and 28% of studies (*n* = 7) did not address whether ethical considerations within the study design or analytic process were described to participants. Regardless, most of the other appraisal criteria were met and therefore the studies were deemed strong enough for inclusion in the present review.

### 3.3 Theoretical framework

There was no universal theoretical framework used by researchers (Table 3). In total, 24 different theoretical frameworks were used within the examined articles to ground methodology and subsequent data analysis, with the most frequently used frameworks being Sexual Stigma and Prejudice Theory [*n *= 5; e.g., (19, 49)] and Multilevel Framework [*n* = 3; e.g., (46, 57)]. Five studies were grounded in theories related to feminism: specifically, Queer Feminist Theory [*n* = 2; e.g., (58, 59)] Black Feminist Theory [*n* = 1; e.g., (60, 61)] Feminist Standpoint Theory [*n* = 1; e.g., (62)] and the Theory of Feminism [*n* = 1; e.g., (63)]. Nine studies did not clearly define the theoretical framework utilized for analysis. Eight studies within the sample leveraged multiple frameworks simultaneously to guide empirical research.

### 3.4 Subject focus, sample, and results

To develop a holistic perspective of sport leaders’ experiences with LGBTQ+ topics, the systematic review of articles was divided into three topic areas prior to the search process. Given the lack of research on LGBTQ+ sport manager experiences (1) and the influence of sport leader policies and practices on inclusion and LGBTQ+ athlete experiences (7, 18, 29), topic areas were pre-selected to best represent the experiences, behaviors, and attitudes of sport leaders in relation to LGBTQ+ topics. More specifically, these subjects included: (a) studies examining the *first-hand lived experiences* of sport leaders with LGBTQ+ sexual identities; (b) studies exploring *stakeholder attitudes* toward LGBTQ+ identifying sport leaders; and (c) studies concerning attitudes of those working within sport leadership positions *towards* LGBTQ+ sport issues. All included articles were analyzed for subject area and sample characteristics to reveal the research focus alongside key findings within the population of interest, as outlined in Table 3.

#### 3.4.1 First-hand lived experiences of LGBTQ+ sport leaders

Twelve studies within the sample examined the first-hand lived experiences of LGBTQ + individuals occupying sport leadership positions. Lesbian females composed the entire sample (*n* = 8) or the majority of the sample (*n* = 2) in 83.3% of studies. Only two studies (23, 24) investigated the experiences of gay men working in sport. Sport coaches were the predominant focus within this subject area, as 58.3% of studies solely examined coach experiences (*n* = 7). Three studies (21, 23, 64) had mixed samples of sport coaches and other employees and two studies (24, 25) did not reveal specific occupational roles to maintain participant confidentiality. Additionally, 66.7% of studies (*n* = 8) had samples in which the majority of participants were White; only one study (21) possessed a sample with majority non-White participants, as Black intercollegiate sport employees comprised most of their sample. Three studies (16, 23, 37) did not provide demographic information to protect participant confidentiality.

Understanding and exploring sexual identity within the context of sport was a major aim of studies examining lived experiences of LGBTQ+ sport leaders. Participants’ experiences were highlighted by the intersectionality of power dynamics, occupational status (64), and social identity (21). Many LGBTQ+ sport leaders reported encountering sexual prejudice or homophobia within their sport organizations or via interactions with colleagues due to their marginalized sexual orientation (13, 15, 16).

In general, sexual identities were classified as complex and fluid in nature. Participants described their “level of outness,” in terms of public disclosure of their sexual orientation, to be dependent on contextual factors such as situational safety, personal comfort level, and an opportunity to foster interpersonal connection (16, 22). Further, LGBTQ+ sport employees and coaches engaged in a variety of identity performance (22, 24) and identity management (25, 37) tactics in occupational settings. For some sport leaders, identity management involved covering [i.e., concealment of stigmatized identity by promoting hyperfeminine or hypermasculine dress or behavior (16, 23)]. Other LGBTQ+ sport leaders compartmentalized their personal lives from their professional lives to conceal their marginalized identity (48). An additional method of covering involved emphasizing athletic and/or occupational identities to be most important to their sense of self, especially when compared to their aspects of themselves within the workplace (24, 25). Together, these covering strategies effectively disguised participants’ marginalized sexual orientation from student-athletes, colleagues, and supervisors alike.

Continuous engagement in identity management practices had differential effects on LGBTQ+ sport leaders. Some lesbian coaches described their workplace as a homophobic environment and feared negative backlash due to their sexual identity (13, 15, 16). Other lesbian coaches felt that identity management provided them with agentic control over their personal narratives. Further, by choosing when and how to disclose their LGBTQ+ identity, some participants were able to engage in radical normalization (22, 37). Iannotta and Kane (22) reported that lesbian coaches strategically normalized their marginalized identity by integrating routine actions, such as casually mentioning their partners during interactions with their athletes and colleagues. Coaches also shared their LGBTQ+ family narratives in their online biographies to publicly normalize their sexual identities (37). These actions aimed to disrupt the heteronormative culture of sport (14), while also promoting inclusion within participants’ respective sport environments.

In the particular case of participants who identified as gay men, these individuals did not always perceive workplace environments in sport as overtly hostile (23, 24). However, their perceptions still influenced their identity management behaviors, in the sense that some perceived their occupational identity to be more central to their person, and therefore downplayed marginalized identities [e.g., sexual orientation (24)]. Consequently, in some cases, gay men who held low occupational status in their organizations (e.g., early career roles, shorter tenure within an organization) concealed their sexual identities by avoiding discussions of their personal lives or “passing” as heterosexual through hypermasculine appearances or behaviors (23).

Coaches and administrators with multiple marginalized identities faced greater pressures to negotiate aspects of their identity to remain working in sport. Walker and Melton (21) reported that lesbian intercollegiate sport employees not only felt they had to manage their marginalized sexual orientation but also their gender and racial identities within their respective athletic departments. Black lesbian employees described that working in collegiate sport was more challenging for them because they consistently needed to manage both their race and sexual orientation without any shared community for their identities (i.e., a Black lesbian community in sport). As such, the necessity to continuously engage in identity management created a tipping point, influencing many participants to leave their occupation (21).

Overall, studies examining the lived experiences of LGBTQ+ individuals in sport leadership positions revealed a predominant focus on lesbian women, especially in coaching roles, with limited representation and exploration of gay men or LGBTQ+ individuals with multiple marginalized identities (e.g., race). Many LGBTQ+ sport leaders reported experiences of encountering homophobia within their workplaces. Identity management strategies varied among participants, ranging from covering, compartmentalization, and radical normalization. Each strategy had a differential effect on participants depending on their personal context (64), and social identities were navigated differently depending on interpersonal, sociocultural, organizational, and environmental factors (21, 23, 48).

#### 3.4.2 Studies exploring stakeholder attitudes toward LGBTQ+ sport leaders

There were limited studies (*n* = 5) in the sample that explored stakeholder attitudes toward LGBTQ+ sport leaders. In these articles, prominent stakeholders included parents (11, 47) and athletes (10, 40, 41). Participants in four studies were predominantly White, with only one study possessing a sample of diverse racial identities [i.e., African American, Hispanic, and White individuals (47)]. Stakeholders identified as predominantly heterosexual in three studies (10, 41, 47); remaining studies did not report the sexual orientation of participants.

Studies explored attitudes of sexual prejudice toward LGBTQ+ sport coaches, as well as affinity for teams led by gay or lesbian coaches. Findings related to parent attitudes were variable: Sartore and Cunningham (10) revealed that parents possessed prejudicial attitudes toward gay and lesbian sport coaches and would not allow their children to compete for them, while Cunningham and Melton (47) found that parents generally possessed positive attitudes toward LGBT coaches, but the level of support varied between unequivocal (e.g., unconditional), indifferent, and qualified (e.g., conditional). Notably, racial identity was classified as a moderating variable in the relationship between parent prejudice and LGB coaches (11).

Similar to parents, athletes also exhibited different attitudes toward LGBTQ+ coaches. Some had more positive views of gay or LGBT-ally prospective coaches (41); others exhibited attitudes of sexual prejudice towards gay or lesbian coaches and, in some cases, conveyed that they would not play for them (10). Gender of the athlete also influenced affinity for teams led by a gay or lesbian coach. Specifically, former male athletes were increasingly influenced to like a team when they were aware that the coach was gay or lesbian (40).

In summary, while two key sport stakeholder groups were studied (parent and athlete), findings regarding attitudes toward LGBTQ+ sport coaches were inconsistent, and at times, contradictory. There was limited information about the influence of demographic variables on stakeholder attitudes, and no research concerning attitudes towards LGBTQ+ individuals in sport leadership positions outside of coaches.

#### 3.4.3 Studies related to sport leader attitudes toward LGBTQ+ sport topics

The majority of articles (*n* = 17) within the sample focused on sport leader attitudes toward LGBTQ+ topics in sport. Across the examined studies in this subject area, participants represented a range of occupational roles and perspectives toward LGBTQ+ topics in sport. Specific occupations included athletic administrators and sport directors (32, 44), high school coaches (17, 35), college coaches (38, 39, 42, 52, 53), sport information directors (36, 45), and athletic departments (46, 54). Studies in this subject area also examined a variety of topics, including attitudes towards LGB sexual identities (*n* = 5), attitudes towards LGB athletes (*n* = 5), inclusiveness (*n* = 3), public same sex family narratives (*n* = 2), and efficacy to coach LGB athletes (*n* = 2). Two primary themes identified across these topic areas were (a) attitudes toward LGBTQ+ student-athletes, and (b) the portrayal and discussion of LGBTQ+ sexual identity in sport, both of which are expanded upon in the following paragraphs.

Sport directors and coaches at both the high school and college levels were surveyed in studies concerning sport leader attitudes toward LGBTQ+ athletes. Antecedents to coach attitudes included education surrounding topics of sexual or gender identities, previous contact with LGBTQ+ athletes, societal influences, age, and religious beliefs (35, 52). Some research suggested that coaches possessed a more tolerant or increasingly positive view toward LGB athletes in Italy (32), China, Taiwan, Hong Kong (52), and the United States (38, 39), which could result in fewer instances of openly prejudicial behaviors based on athlete sexual identity (32, 52). Other studies identified heterosexist norms to be prevalent amongst coaches (51) and other sport leaders, including sport information directors (45); these studies indicated that structural ideologies (i.e., gender ideology, heterosexist ideology) continue to influence individual attitudes and thoughts toward LGBTQ+ topics (17, 39).

The influence of heterosexist ideology extended beyond mere attitudes and thoughts to impact sport leader behaviors. This was specifically observed in the context of LGBTQ+ identities. Particularly, coaches at both the high school (17) and college (33, 54) levels did not openly discuss LGBTQ+ topics with their athletes. Instead, coaches failed to acknowledge the sexual orientation of LGBTQ+ athletes (32) or chose to prioritize the acknowledgment of their athletic identities over other identities such as sexual orientation (52). Further, organizational sport managers (50), intercollegiate athletic administrators (44), and coaches (42) felt like they lacked knowledge and/or experience navigating LGBTQ+ sport issues or access to appropriate resources to do so. The lack of acknowledgement and education surrounding the presence of LGBTQ+ sexual orientations demonstrated a form of covert silencing that perpetuated heterosexism (17, 32).

Silencing of LGBTQ+ sexual orientations by sport leaders also extended to overt forms across included studies. For example, by mere association with LGBTQ+ identities (e.g., being labeled or perceived as a “lesbian”), both heterosexual and queer female coaches engaged in specific occupational behaviors (i.e., staff hiring practices, student-athlete recruitment strategies) to ensure that they avoided the “lesbian” label while navigating their career paths (53). Understanding the potential repercussions such a label could pose to their career progression in a new institution, coaches also reported altering their career choices (i.e., accepting new positions) to avoid these perceived or actual labels (53). Additionally, sport information directors in collegiate athletic departments engaged in gatekeeping practices by excluding same sex family narratives in public online coaching biographies (33, 34) while reporting their belief that they did not view homophobia as an issue in intercollegiate athletics (36).

While some participants within the included studies displayed increasing tolerance toward LGBTQ+ athletes, the research within this subject area indicates that heterosexism and heteronormativity continue to exist at a structural level across sport organizations. Heterosexist ideology influenced sport leader attitudes, thoughts, and behaviors. It resulted in the covert silencing of LGBTQ+ identities and continued presence of organizational barriers (such as exclusionary gatekeeping), all of which underscore the complexities of LGBTQ+ inclusion within sport.

## 4 Discussion

The sexual orientation of sport leaders influences not only the ways in which they experience their lives but also how they experience their occupational role and environment (9, 15, 25). This systematic review found that sport leaders, including coaches and athletic administrators, continue to face marginalization because of heteronormativity and homophobia (10, 20, 22). This marginalization was present in everyday interactions (13) and was upheld by leaders and organizational practices (14, 21, 64). These findings align with extant scholarship in relation to LGBTQ+ discrimination in sport (3, 7).

The predominant theme across included studies underscored a heterosexist notion: sport is a place where sexual orientation should not be present nor discussed (17, 18, 32, 45). Exacerbated by a lack of knowledge surrounding inclusive LGBTQ+ practices, policies, and resources (17, 39, 42, 44), coaches and administrators perpetuate this notion in their organizational cultures. These practices do not only impact athletes, but also LGBTQ+ identifying sport leaders and sport leader attitudes towards LGBTQ+ issues. In essence, the research findings underscore the need to further explore and measure allyship behaviors in sport leadership positions (30), and the occupational behaviors of LGBTQ+ sport leaders.

The review of the included studies indicates that many sport organizations operate inclusively out of compliance. Additionally, previous literature denotes that leaders can react *ad hoc* to avoid legal repercussions (27), rather than proactively fostering LGBTQ+ inclusion through practices and policies (18). Management by this philosophy can result in the differential treatment of LGBTQ+ individuals in sport settings, whether by policies (e.g., specific team or departmental rules) or institutionalized practices [e.g., “Don't Ask, Don't Tell” behaviors (28)]. When sport leaders do attempt to proactively address LGBTQ+ inclusion (42), they must balance competing interests within and beyond their organizations. This creates additional barriers to fostering inclusion, especially if leaders are fearful of losing the support from external stakeholders [e.g., athletic donors, boosters, and sponsors (18)].

Some research suggests that sport is becoming more LGBTQ+ inclusive. LaVoi et al. (37) noted an increase in the visibility of openly lesbian coaches in public online biographies, and Scheadler et al. (41) revealed increasingly positive athlete attitudes towards LGBT+ or LGBT-ally coaches. However, LaVoi et al. (37) reported only 0.5% of examined online biographies contained a same-sex family narrative. Further, Scheadler et al. (41) described that some athletes viewed sexuality as a weakness, and others expressed respect for an anti-LGBT coach's views.

However, multiple studies in this systematic review present interpretive paradoxes for LGBTQ+ identities and topics in sporting contexts. Particularly, sport employees value LGBTQ+ inclusion in the workplace (46), yet sport leaders fail to portray same sex family narratives or believe that LGBTQ+ identities should not be displayed publicly (36, 45); LGBTQ+ sport leaders recognize the importance of their sexual identity (37), yet they engage in a variety of identity management techniques to conceal it (16, 23); sport leaders report more positive attitudes toward LGBTQ+ individuals (38, 52), yet they reproduce heterosexism in policy and practice (21, 39); sport environments are viewed as less hostile for LGBTQ+ identifying individuals because of a decrease in overt homonegativity (24), yet more subtle forms of discrimination, like ignoring or the silencing of LGBTQ+ identities, equally communicates exclusion (14, 32).

The results of these studies convey that while inclusion may be championed on an interpersonal level (64), it faces sociocultural and structural barriers at large due to the heteronormative and heterosexist nature of sport (14, 47). Inclusion can serve as an umbrella term that sport leaders use to promote ideological progression, but in reality, it is not practiced. Instead, sport leaders fail to regularly acknowledge LGBTQ+ sexual orientations in athletic contexts for athletes, coaches, and other stakeholders. By avoiding this, they fail to bridge the connection between identity and behavioral practice in sport leadership.

### 4.1 Strength and weaknesses

The studies in this review possess several strengths. The first is the use of multiple theoretical frameworks to guide methodology and data analysis in qualitative, quantitative, and mixed method studies. The lack of a universally accepted framework within this subject area emphasizes the need to understand LGBTQ+ experiences in sport from multiple perspectives. Additionally, it provides both practitioners and scholars with a complex, nuanced understanding of LGBTQ+ experiences and topics from a sport leadership perspective.

The second is the use of qualitative methodologies throughout strictly qualitative research and in mixed method studies. Qualitative research can spotlight the voices and experiences of marginalized individuals. It also provides participants the opportunity to not only describe the what (e.g., behaviors, attitudes, experiences, beliefs) but also the how or why (e.g., explaining attitudes, events, experiences) in greater detail to reveal contradictory perceptions *towards* and the complexity *of* lived experiences for LGBTQ+ sport leaders.

The third is the examination of sport leaders’ attitudes toward LGBTQ+ topics from a variety of occupational positions, including high school coaches, college coaches, athletic directors, sport administrators, and sport information directors, among others. Including participants from multiple sport leadership positions allowed for a comprehensive understanding of their attitudes and beliefs toward LGBTQ+ inclusion.

It is also important to note limitations of included studies. First, only one study (44) in this review examined sport leader attitudes towards transgender inclusion. Policies and provisions surrounding transgender athlete participation create repercussions for all transgender, nonbinary, gender non-conforming and/or intersex individuals operating in sport, including sport leaders. As such, future research must address the experiences of all athletes and sport leaders encompassed by the LGBTQ+ acronym.

Second, the majority of studies that reported racial demographic information (*n *= 12) had a participant sample that was predominantly White. While this is reflective of current demographic data for sport leaders (i.e., coaches, athletic directors, athletic administrators) across NCAA divisions (65), it fails to include the perspectives of Black, Asian, Hispanic, Latino, Multiracial, and additional marginalized racial identities who also identify as LGBTQ+. Only one study examined the experiences of current and former sport employees with multiple marginalized identities (21).

Third, consistent with previous literature (2), identity was the most prevalent topic explored when considering the firsthand experiences of LGBTQ+ sport leaders. Further, in this investigation of lived experiences, examined studies contained homogeneous samples in terms of gender and occupational role. Many studies (*n* = 11) had samples composed of predominantly female participants; additionally, most studies (*n* = 16) explored the experiences or attitudes of sport coaches. Only two articles (23, 24) explored the lived experiences of gay men. Outside of two studies with mixed coach-administrator samples (21, 64) or athletic department case studies (25, 46, 54), no studies examined the experiences of athletic administrators, who serve as major sport leaders in intercollegiate athletic departments in the U.S. Because participant samples were similar in terms of occupation and gender, it was not possible to draw comprehensive conclusions about the diverse experiences of LGBTQ+ sport leaders.

Fourth, the most frequently researched LGBTQ+ topic in sport leadership was sport leader attitudes toward LGBTQ+ topics (*n* = 17). However, a closer examination of topic areas revealed that only one study explored administrative decision-making related to organizational policies and practices (44). This gap in the literature is interesting, considering that administrative functions involving planning, implementation, and evaluation of policies and practices are key occupational responsibilities for athletic administrators, especially at the collegiate level.

### 4.2 Future directions

This systematic review critically explored findings related to lived experiences of LGBTQ+ sport leaders, attitudes toward LGBTQ+ sport leaders, and attitudes of sport leaders toward LGBTQ+ topics. A comprehensive picture of the extant scholarship shows that sport leadership positions at large are still understudied, especially in the context of LGBTQ+ identities and topics.

Future studies can extend previous research by: (a) continuing to explore the experiences of LGBTQ+ athletic administrators, with particular emphasis on demographics that are historically (and remain) underrepresented in sport scholarship, including the experiences of bisexual people and gay men, transgender and/or gender-nonconforming individuals, as well as Black, Asian, Hispanic, Latino, Multiracial, and individuals with other marginalized racial identities; (b) continuing to examine the intersectional experiences of LGBTQ+ sport leaders (e.g., race, gender, sexual orientation); (c) interrogating the privilege and influence of occupational status and power in conjunction with marginalized sexualities in sport leadership positions; (d) investigating the leadership behaviors of LGBTQ+ athletic administrators in relation to decision-making; (e) surveying the attitudes of athletic administrators toward LGBTQ+ coaches and/or topics [see (30)]; (f) investigating the attitudes of sport leaders toward transgender sport policies and inclusion at all organizational and competitive levels; and (g) examining (with the intent to reform) the effectiveness of current educational trainings/programming surrounding LGBTQ+ identities and topics.

It is important to acknowledge the existence of significant sociopolitical barriers that can impact scholarship. One such example is in the United States. As of 2024, 85 legislative bills that prohibit DEI initiatives and training in admissions, employment, and/or education regarding race, ethnicity, national origin, sexual orientation, gender identity, gender expression, and religion have been introduced to U.S. Congress. In 13 states (Idaho, Wyoming, North Dakota, Utah, Texas, Kansas, Indiana, Tennessee, Alabama, North Carolina, and Florida), these bills have been signed into law (66). Further, each of these proposed or passed bills invoke nuanced impacts due to various prohibitions. Anti-DEI legislation could have a chilling effect on scholarship in this area due to reduced institutional support, decreased research funding, and the existence of potential occupational and physical dangers to the safety of researchers and participants. At the time of this manuscript submission, the consequences of these legislative bills on DEI-related scholarship in sport have not been studied.

Beyond legislation, sociocultural barriers in sport remain—resulting in the stigmatization of and discrimination against LGBTQ+ individuals working and participating in sport (7). These barriers impact the lived experiences of LGBTQ+ sport leaders and pose significant challenges for researchers attempting to study this population. If LGBTQ+ individuals cannot safely disclose their identities in the workplace, it is difficult for researchers to identify and recruit participants to conduct studies.

To advance this research, exploring future avenues of scholarship is needed, especially in light of the aforementioned barriers. To best promote the visibility of LGBTQ+ research, it is crucial to collaborate with scholars and practitioners to highlight the importance of LGBTQ+ allyship/advocacy in sport settings and to promote policies that protect DEI initiatives in sport and other social institutions.

## Data Availability

The original contributions presented in the study are included in the article/Supplementary Material, further inquiries can be directed to the corresponding author.

## Footnotes

^1^The term “LGBTQ+” was used throughout this manuscript to mirror language in reviewed studies. The authors reference specific labels within the acronym “LGBTQ+” that were also explicitly represented in included studies. To the authors’ best knowledge, intersex and asexual identities were not represented in this sample.
